# Abscopal effect of radiation on lung metastases of hepatocellular carcinoma: a case report

**DOI:** 10.1186/1752-1947-5-111

**Published:** 2011-03-19

**Authors:** Kae Okuma, Hideomi Yamashita, Yuzuru Niibe, Kazushige Hayakawa, Keiichi Nakagawa

**Affiliations:** 1 Department of Radiation Oncology, University of Tokyo Hospital, 7-3-,1 Hongo, Bunkyo-ku, Tokyo 113-8655, Japan; 2 Department of Radiation Oncology, Kitasato School of Medicine, 1-15-1, Kitasato, Minami-ku, Sagamihara, Kanagawa 252-0375, Japan

## Abstract

**Introduction:**

The abscopal effect is the effect of radiation therapy at a site distant to the area of irradiation. This is not a common event and has not been clearly defined, resulting in few reported cases in the literature. We discuss this phenomenon in a patient with hepatocellular carcinoma.

**Case presentation:**

A 63-year-old Japanese man underwent extended right hepatic lobectomy for hepatocellular carcinoma. During his follow-up examination, a single lung metastasis and a single mediastinal lymph node metastasis were found. Trans-catheter arterial embolization was initially attempted to treat the mediastinal tumor, however this approach failed to take effect and carried risks of spinal artery embolism. External-beam irradiation, with a dose of 2.25 Gy per fraction, was performed using an antero-posterior parallel-opposed technique (total dose, 60.75 Gy). A computed tomography scan performed one month after starting radiotherapy showed a remarkable reduction of the mediastinal lymph node metastasis. In addition to this, we observed spontaneous shrinking of the lung metastasis, which was located in the right lower lobe and out of the radiation field. No chemotherapy was given during the period. There has been no recurrence of either the lung metastasis or the mediastinal lymph node metastasis during a follow-up 10 years after the radiotherapy.

**Conclusion:**

We observed a rare abscopal effect in a site distant from the area of irradiation. Irradiation of the mediastinum resulted in tumor mass regression in the untreated lung tumor.

## Introduction

An abscopal effect has been defined as a reaction outside an irradiated area but within the same organism [1], that can result in a tumor in a non-irradiated area being spontaneously reduced. Since the first report of an abscopal effect by Mole in 1953 [2], several other cases have been reported in malignant lymphoma [3-5], hepatocellular carcinoma (HCC) [6] and malignant melanoma [7]. In 2007, Takaya *et al*. described an abscopal effect in a case of toruliform para-aortic lymph node metastasis in a patient with advanced uterine cervical carcinoma. This patient was treated with external whole-pelvis and intra-cavitary irradiation to the primary pelvic lesion, successfully resulting in disappearance of the lesion. Moreover, para-aortic lymph node metastases outside the irradiated field also spontaneously disappeared [8].

The mechanism of the abscopal effect has not been clearly defined. We report here the case of a patient who showed an abscopal effect on lung metastases of HCC.

## Case presentation

**Figure 1:**
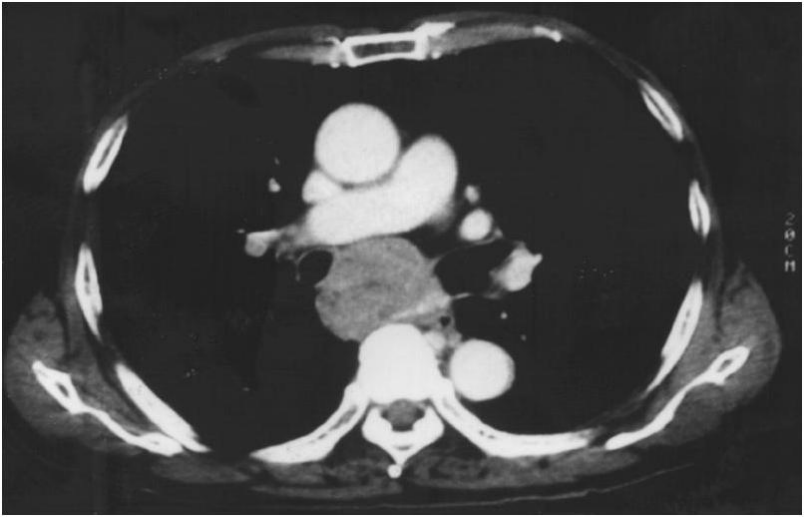
**Chest CT image before radiation therapy**. A bulky mediastinal lymph node metastasis was detected.

**Figure 2:**
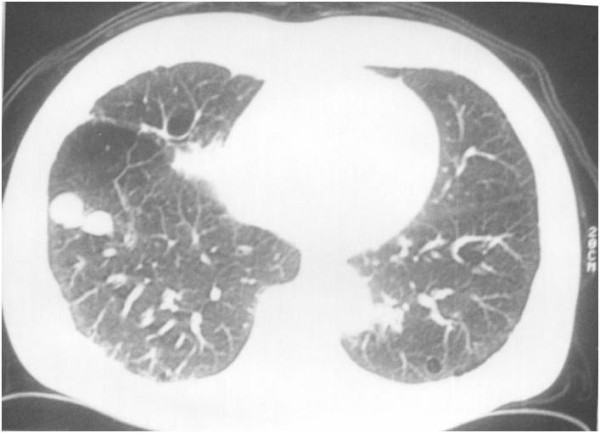
**Two lung metastases**.

A Japanese man, who had been followed since 53 years of age by the respiratory department of our Medical Center due to bronchial asthma, was hospitalized for progression of asthma at 63 years old. A suspected diagnosis of HCC in his right liver lobe was confirmed by abdominal computed tomography (CT). An extended right lobectomy was performed after three months in our hospital. Pathologic examination revealed an HCC, composed of a necrotic tumor that measured 10.5 × 9 × 11 cm. In addition, there were three daughter nodules with diameters of less than 1 cm each. The residual nodular tumor without necrosis was Edmondson grade II to III with nuclear atypia, and was moderately differentiated. Invasion of lymphatic and vascular channels was not obvious. Exposure to the surface of the liver capsule was not found. A single lung metastasis and a single mediastinal lymph node metastasis were found in a chest CT scan performed 18 months after the liver lobectomy (Figures 1 and 2). Final diagnosis was made based on the elevated levels of two tumor markers for HCC: α-fetoprotein (AFP) (4869 ng/mL) and protein induced by vitamin K absence or antagonists II (PIVKA-II) (>20,000 mAU/mL). Trans-catheter arterial embolization for the mediastinal tumor was attempted, however the risk of spinal artery embolism resulted in the decision to only examine the tumor at that time.

**Figure 3:**
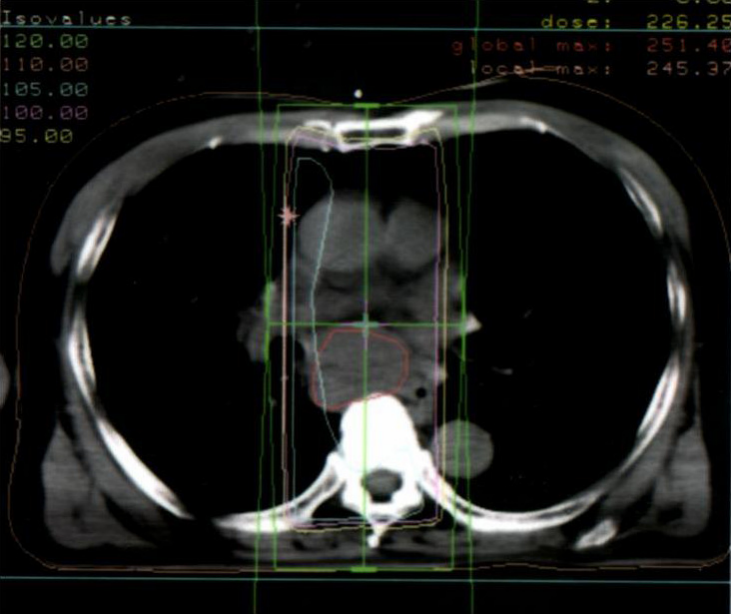
**Chest CT image showing radiation dose distribution: axial view**.

**Figure 4:**
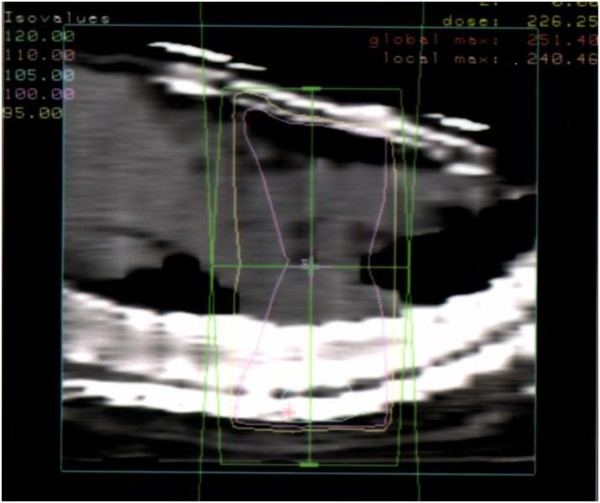
**Chest CT image showing the radiation dose distribution: sagittal view**.

**Figure 5:**
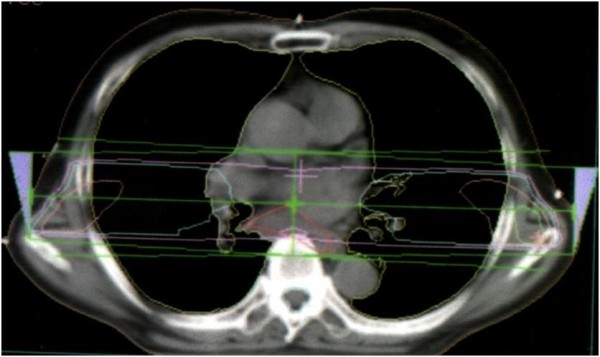
**Chest CT image showing radiation dose distribution after 40 Gy**.

**Figure 6:**
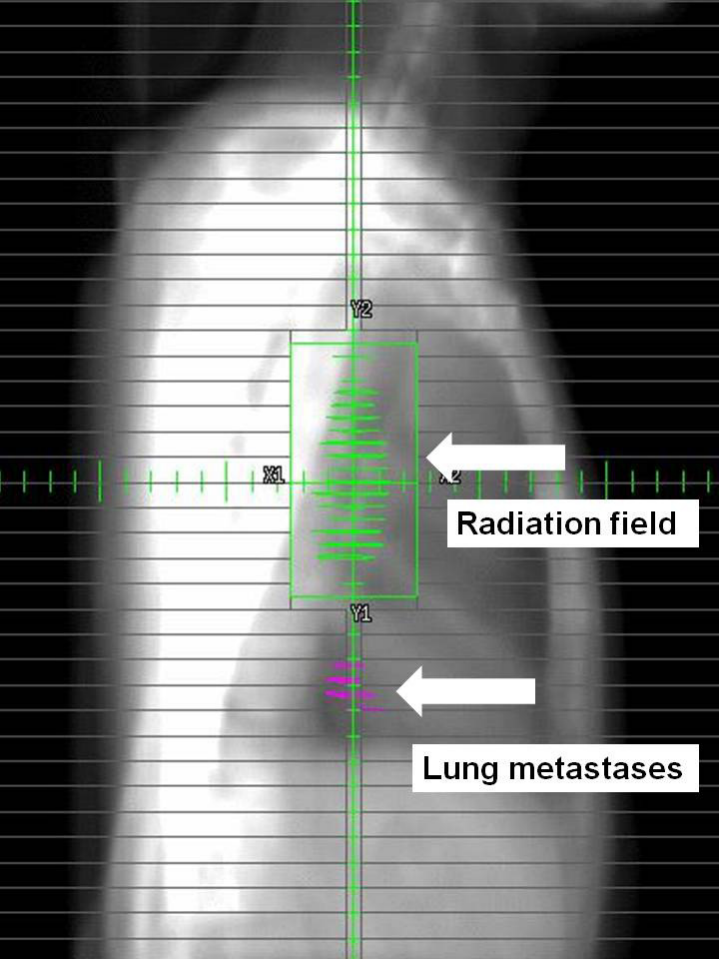
**Beam's eye view of the lateral beam of the radiation therapy**. This shows the metastatic lung tumor is outside the target area.

**Figure 7:**
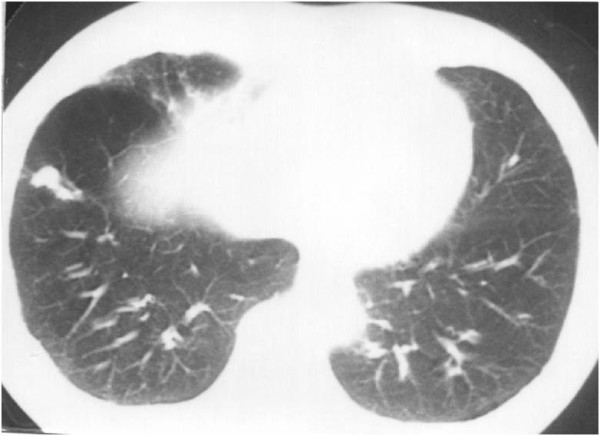
**Chest CT scan after radiation therapy**. Both lung metastases had shrunk under radiation therapy.

**Figure 8:**
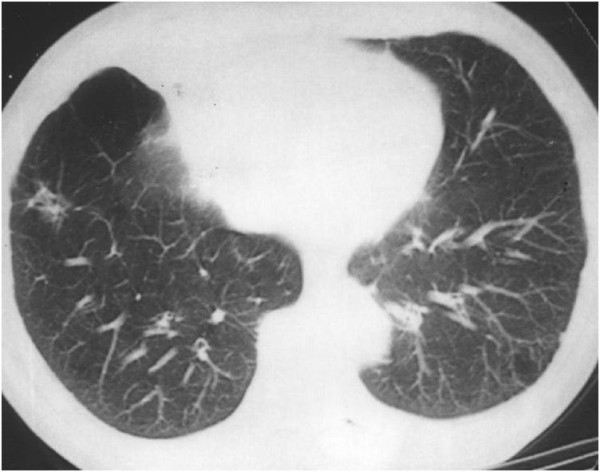
**CT scan showing the absence of both lung metastases, which regressed after radiation therapy**.

Our patient was admitted to our Department of Radiation Oncology for irradiation. His Karnofsky performance status score was 90% to 100%. He complained of a moderate cough and moderate bloody sputum but denied any dyspnea or chest pain. To relieve these clinical symptoms, external-beam irradiation, with a dose of 2.25 Gy per fraction, was performed using an antero-posterior parallel-opposed technique (total dose, 60.75 Gy) (Figures 3 and 4). The energy was 10 megavolts. The radiation field was set for gross tumor volume plus a margin of 1 cm. The field size was 8 × 10 cm. Radiation therapy was given four times a week. After 40 Gy, the radiation field was changed to left-right parallel-opposed beams to spare the spinal cord (Figure 5). The lung metastasis that had induced no clinical symptoms was not treated with radiation therapy, and was located outside the radiation field of the left-right opposing beams (Figure 6). A CT scan was performed after the radiation therapy, which showed a remarkable reduction in the mediastinal lymph node. Additionally, shrinking of the tumor in his right lower lobe, outside of the radiation field, was observed (Figures 7 and 8). No chemotherapy had been given during this period.

During follow-up as an out-patient, our patient was observed to have dyspnea (Hugh-Jones 1-2), slight cough and slight sputum. AFP levels had decreased to 23 ng/mL, and PIVKA-II to 13 mAU/mL.

A CT scan performed four years after the radiation therapy showed a lymph node swelling with a diameter of 3.5 cm in the area of origin of the left gastric artery. At this time, AFP and PIVKA-II were elevated to 1990 ng/mL and 1990 mAU/mL respectively, but with no pathologic evidence of recurrence. Stereotactic body radiotherapy for the lesion was performed, with 30 Gy in three fractions. The recurrent tumor disappeared. Six and a half months after the stereotactic body radiotherapy, no obvious recurrent disease was found.

## Discussion

Many case reports describing spontaneous regression of HCC can be found in the literature. Since 1982, about 60 cases have been reported as abscopal effects or spontaneous regressions. These cases were reviewed by Oquiñena *et al*. in 2009 [9]. Although many similar reports have been published [3,4,6-8,10], the abscopal effect is still a rare phenomenon, and the mechanism has not been clearly defined. Lin *et al*. proposed two mechanisms involving ischemia and an immune response [11]. First, mitotic inhibitors (cytokines) induced by local radiation are released into the circulation and mediate a systemic anti-tumor effect. This hypothesis is supported by reports that circulating tumor necrosis factor levels are elevated after radiotherapy, and have coincided with the regression of an HCC situated away from the radiation field [2,12]. Second, irradiation of a tumor in one site induces the release of circulating tumor antigen or inflammatory factors, which may then mediate an augmented immune response against non-irradiated, malignant lesions expressing similar tumor antigens. It has previously been shown that local radiotherapy increases the activity of natural killer cells [13,14]. Dewan *et al*. presented the hypothesis that the type of dose fractionation regimen determines the ability of radiotherapy to synergize with anti-CTLA-4 antibody [15]. Although these hypotheses have some merit, at present they remain to be confirmed.

## Conclusion

We observed a rare abscopal effect in a site distant from the area of irradiation. Irradiation of a tumor in the mediastinal resulted in tumor mass regression in an untreated lung metastasis of HCC.

## Consent

Written informed consent was obtained from the patient for publication of this case report and any accompanying images. A copy of the written consent is available for review by the Editor-in-Chief of this journal.

## Competing interests

The authors declare that they have no competing interests.

## Authors' contributions

KO undertook the gathering of information for this case and was a major contributor in writing the manuscript. HY conceived the manuscript and was a major contributor to the manuscript. All authors read and approved the final manuscript.
